# (*E*)-1-(4,4′′-Difluoro-5′-meth­oxy-1,1′:3′,1′′-terphenyl-4′-yl)-3-phenyl­prop-2-en-1-one

**DOI:** 10.1107/S1600536811045375

**Published:** 2011-11-02

**Authors:** Richard Betz, Thomas Gerber, Eric Hosten, S. Samshuddin, Badiadka Narayana, Hemmige S. Yathirajan

**Affiliations:** a Nelson Mandela Metropolitan University, Summerstrand Campus, Department of Chemistry, University Way, Summerstrand, PO Box 77000, Port Elizabeth 6031, South Africa; b Mangalore University, Department of Studies in Chemistry, Mangalagangotri 574 199, India; c University of Mysore, Department of Studies in Chemistry, Manasagangotri, Mysore 570 006, India

## Abstract

The title compound, C_28_H_20_F_2_O_2_, is a polysubstituted terphenyl derivative bearing a Michael system. The C=C double bond is *E* configured. In the crystal, C—H⋯O and C—H⋯F contacts connect the mol­ecules, forming undulating sheets that lie perpendicular to the crystallographic *a* axis. The shortest π–π inter­action [centroid–centroid distance = 3.7163 (7) Å] involves the *para*-fluoro­phenyl ring in the *para* position to the Michael system, and its symmetry-generated equivalent.

## Related literature

For the pharmacological importance of terphenyls, see: Liu (2006 ▶) and of chalcones, see: Dhar (1981 ▶); Dimmock *et al.* (1999 ▶); Satyanarayana *et al.* (2004 ▶). For our work on the synthesis of different derivatives of chalcones, see: Samshuddin *et al.* (2011*a* ▶,*b* ▶); Fun *et al.* (2010*a* ▶,*b* ▶); Jasinski *et al.* (2010*a* ▶,*b* ▶); Baktır *et al.* (2011*a* ▶,*b* ▶). For graph-set analysis of hydrogen bonds, see: Etter *et al.* (1990 ▶); Bernstein *et al.* (1995 ▶).
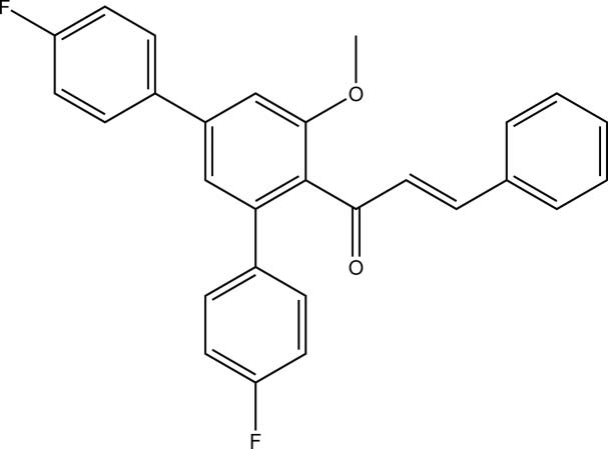


## Experimental

### 

#### Crystal data

C_28_H_20_F_2_O_2_
*M* *_r_* = 426.44
Monoclinic, 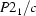

*a* = 13.9226 (4) Å
*b* = 6.7977 (2) Å
*c* = 22.4531 (7) Å
β = 101.874 (1)°
*V* = 2079.53 (11) Å^3^
*Z* = 4
Mo *K*α radiation
μ = 0.10 mm^−1^
*T* = 200 K
0.48 × 0.13 × 0.10 mm

#### Data collection

Bruker APEXII CCD diffractometer
19250 measured reflections
5125 independent reflections
3527 reflections with *I* > 2σ(*I*)
*R*_int_ = 0.040

#### Refinement

*R*[*F* ^2^ > 2σ(*F* ^2^)] = 0.037
*wR*(*F* ^2^) = 0.094
*S* = 1.01
5125 reflections
290 parameters
H-atom parameters constrained
Δρ_max_ = 0.23 e Å^−3^
Δρ_min_ = −0.21 e Å^−3^

### 

Data collection: *APEX2* (Bruker, 2010 ▶); cell refinement: *SAINT* (Bruker, 2010 ▶); data reduction: *SAINT*; program(s) used to solve structure: *SHELXS97* (Sheldrick, 2008 ▶); program(s) used to refine structure: *SHELXL97* (Sheldrick, 2008 ▶); molecular graphics: *ORTEP-3* (Farrugia, 1997 ▶) and *Mercury* (Macrae *et al.*, 2008 ▶); software used to prepare material for publication: *SHELXL97* and *PLATON* (Spek, 2009 ▶).

## Supplementary Material

Crystal structure: contains datablock(s) I, global. DOI: 10.1107/S1600536811045375/su2328sup1.cif

Structure factors: contains datablock(s) I. DOI: 10.1107/S1600536811045375/su2328Isup2.hkl

Supplementary material file. DOI: 10.1107/S1600536811045375/su2328Isup3.cdx

Supplementary material file. DOI: 10.1107/S1600536811045375/su2328Isup4.cml

Additional supplementary materials:  crystallographic information; 3D view; checkCIF report

## Figures and Tables

**Table 1:** Hydrogen-bond geometry (Å, °)

*D*—H⋯*A*	*D*—H	H⋯*A*	*D*⋯*A*	*D*—H⋯*A*
C25—H25⋯O1^i^	0.95	2.41	3.3092 (15)	157
C44—H44⋯F2^ii^	0.95	2.55	3.2761 (15)	133

Symmetry codes: (i) 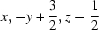; (ii) 
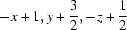
.

**Fig. 1.**
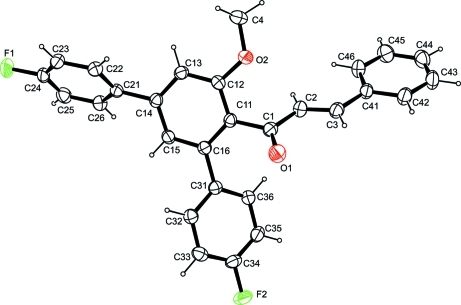
The molecular structure of the title compound, with atom labels and displacement ellipsoids drawn at the 50% probability level.

**Fig. 2.**
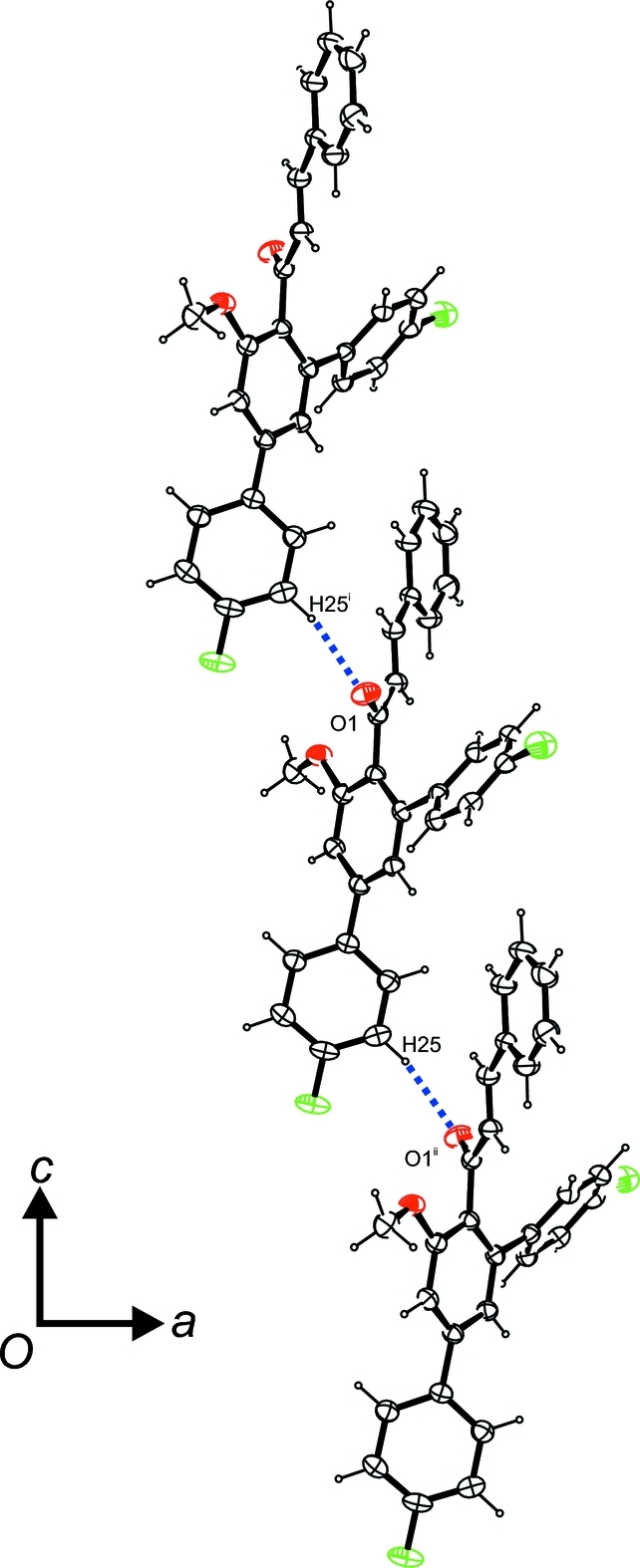
Intermolecular C–H···O contacts, viewed along [0 1 0]. (Symmetry operators: (i) x, -y + 3/2, z + 1/2; (ii) x, -y + 3/2, z - 1/2; displacement ellipsoids are drawn at the 50% probability level).

**Fig. 3.**
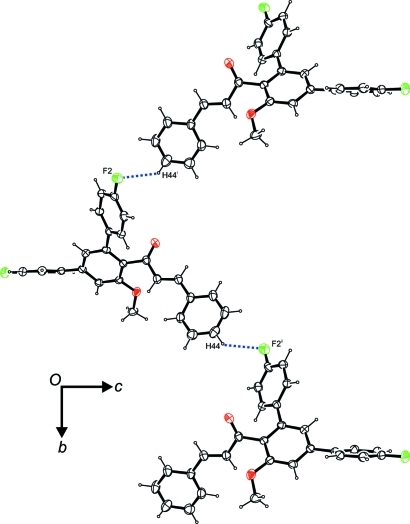
Intermolecular C–H···F contacts, viewed along [-1 0 0]. (Symmetry operators: (i) -x + 1, y - 3/2, -z + 1/2; (ii) -x + 1, y + 3/2, -z + 1/2; displacement ellipsoids are drawn at the 50% probability level).

**Fig. 4.**
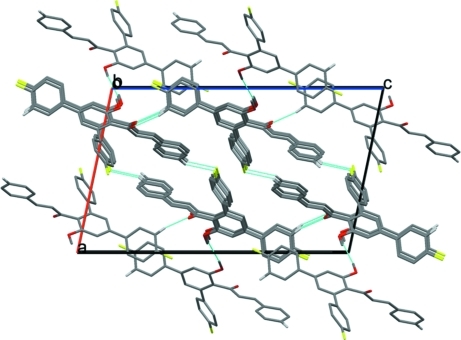
Crystal packing of the title compound, viewed along [0 1 0].

## supplementary crystallographic information

### Comment

Chalcones constitute an important family of substances belonging to flavonoids, a large group of natural and synthetic products with interesting physicochemical properties, biological activity and structural characteristics. They have been reported to possess many interesting pharmacological activities (Dhar, 1981) including anti-inflammatory, antimicrobial, antifungal, antioxidant, cytotoxic, antitumor and anticancer activities (Dimmock *et al.*, 1999; Satyanarayana *et al.*, 2004).In recent years, it has been reported that some terphenyls exhibit considerable biological activities (*e.g.* being potent anticoagulants, immunosuppressants, antithrombotics, neuroprotectives, specific 5-lipoxygenase inhibitors) and showing cytotoxic activities (Liu, 2006). In view of the pharmacological importance of terphenyls and chalcones, and in continuation of our work on synthesis of various derivatives of 4,4'-difluoro chalcone (Samshuddin *et al.*, 2011*a*/*b*, Fun *et al.*, 2010*a*/*b*, Jasinski *et al.*, 2010*a*/*b*, Baktır *et al.*, 2011*a*/*b*), the molecular and crystal structure of the title compound is reported herein.

The molecular structure of the title molecule is shown in Fig. 1. The C═C double bond along the Michael system is (*E*)-configured. The mean planes of the two *para*-fluoro phenyl moieties, (C21-C26) and (C31-C36), enclose angles of 49.66 (5) and 42.33 (5)°, respectively, with the mean plane of the central phenyl ring (C11-C16) in the terphenyl moiety.

In the crystal, C–H···O contacts as well as C–H···F contacts are present (Table 1). While the range of the latter ones falls only by more than 0.1 Å below the sum of van-der-Waals radii of the corresponding atoms, the shortening of the C–H···O contacts is found to be more than 0.3 Å below this cut-off criterion. The C–H···O contacts are apparent between one of the hydrogen atoms, H25, in an *ortho* position to fluorine atom, F1, on one of the *para*-fluoro phenyl moieties and the oxygen atom, O1, of the Michael system. The C–H···F contacts are exclusively supported by the fluorine atom, F2, of the *para* fluoro phenyl moiety that is not part of the system of C–H···O contacts. In terms of graph-set analysis (Etter *et al.*, 1990; Bernstein *et al.*, 1995), the descriptor for the C–H···O contacts is *C*^1^_1_(10) on the unitary level (Fig. 2), while the C–H···F contacts necessitate a *C*^1^_1_(15) descriptor on the same level (Fig. 3). In total, the molecules are connected to form undulating sheets lieing perpendicular to [100].

The crystal packing of the title compound is shown in Figure 4. The shortest π–π centroid-centroid distance is 3.7163 (7) Å, involving the *para*-fluoro phenyl moiety (C21-C26) and its symmetry-generated equivalent (symmetry code: -x, 0.5+y, -0.5-z).

### Experimental

To a mixture of 1-(4,4''-difluoro-5'-methoxy-1,1':3',1''-terphenyl-4'-yl) ethanone (0.338 g, 0.001 mol) and benzaldehyde (0.106 g, 0.001 mol) in 20 mL of ethanol, was added 1 ml of 10% sodium hydroxide solution. the mixture was stirred at 278–283 K for 3 h. The precipitate formed was collected by filtration and dried (yield: 86%). The single-crystal suitable for the X-ray diffraction study was grown from a DMF-ethanol (*v*:*v* 1:1) solution of the title compound by slow evaporation at room temperature.

### Refinement

C-bound H atoms were placed in calculated positions and refined as riding atoms: C—H = 0.95 Å for aromatic and vinylic H atoms, 0.99 Å for methylene and 0.98 Å for methyl H atoms, with U_iso_(H) = k × U_eq_(C), where k = 1.5 for methyl H atoms, and k = 1.2 for all other H atoms.

### Crystal data

**Table:**

C_28_H_20_F_2_O_2_	*F*(000) = 888
*M**_r_* = 426.44	*D*_x_ = 1.362 Mg m^−^^3^
Monoclinic, *P*2_1_/*c*	Melting point: 423 K
Hall symbol: -P 2ybc	Mo *K*α radiation, λ = 0.71073 Å
*a* = 13.9226 (4) Å	Cell parameters from 7468 reflections
*b* = 6.7977 (2) Å	θ = 2.6–28.0°
*c* = 22.4531 (7) Å	µ = 0.10 mm^−^^1^
β = 101.874 (1)°	*T* = 200 K
*V* = 2079.53 (11) Å^3^	Platelet, colourless
*Z* = 4	0.48 × 0.13 × 0.10 mm

### Data collection

**Table:**

Bruker APEXII CCD diffractometer	3527 reflections with *I* > 2σ(*I*)
Radiation source: fine-focus sealed tube	*R*_int_ = 0.040
graphite	θ_max_ = 28.3°, θ_min_ = 1.9°
φ and ω scans	*h* = −18→17
19250 measured reflections	*k* = −8→9
5125 independent reflections	*l* = −25→29

### Refinement

**Table:**

Refinement on *F*^2^	Primary atom site location: structure-invariant direct methods
Least-squares matrix: full	Secondary atom site location: difference Fourier map
*R*[*F*^2^ > 2σ(*F*^2^)] = 0.037	Hydrogen site location: inferred from neighbouring sites
*wR*(*F*^2^) = 0.094	H-atom parameters constrained
*S* = 1.01	*w* = 1/[σ^2^(*F*_o_^2^) + (0.0511*P*)^2^] where *P* = (*F*_o_^2^ + 2*F*_c_^2^)/3
5125 reflections	(Δ/σ)_max_ < 0.001
290 parameters	Δρ_max_ = 0.23 e Å^−^^3^
0 restraints	Δρ_min_ = −0.21 e Å^−^^3^

### Fractional atomic coordinates and isotropic or equivalent isotropic displacement parameters (Å^2^)

**Table:**

	*x*	*y*	*z*	*U*_iso_*/*U*_eq_	
F1	−0.03088 (6)	0.94826 (11)	−0.35137 (3)	0.0494 (2)	
F2	0.50301 (6)	0.12071 (11)	0.05515 (3)	0.0493 (2)	
O1	0.21900 (6)	0.67802 (12)	0.11362 (4)	0.0394 (2)	
O2	0.11107 (6)	1.12741 (12)	0.04508 (4)	0.0353 (2)	
C1	0.23288 (8)	0.83556 (17)	0.09109 (5)	0.0270 (3)	
C2	0.27709 (8)	1.00565 (17)	0.12773 (5)	0.0300 (3)	
H2	0.2950	1.1175	0.1071	0.036*	
C3	0.29307 (8)	1.00990 (18)	0.18816 (5)	0.0310 (3)	
H3	0.2748	0.8965	0.2079	0.037*	
C4	0.06138 (9)	1.30889 (18)	0.02758 (6)	0.0368 (3)	
H4A	−0.0034	1.2818	0.0022	0.055*	
H4B	0.0999	1.3881	0.0045	0.055*	
H4C	0.0536	1.3812	0.0641	0.055*	
C11	0.20575 (8)	0.86471 (16)	0.02319 (5)	0.0238 (2)	
C12	0.13992 (8)	1.01639 (16)	0.00102 (5)	0.0257 (2)	
C13	0.10403 (8)	1.04127 (16)	−0.06077 (5)	0.0267 (2)	
H13	0.0582	1.1431	−0.0749	0.032*	
C14	0.13529 (8)	0.91667 (16)	−0.10215 (5)	0.0253 (2)	
C15	0.20392 (8)	0.77097 (16)	−0.08044 (5)	0.0260 (2)	
H15	0.2276	0.6901	−0.1088	0.031*	
C16	0.23890 (8)	0.74032 (15)	−0.01843 (5)	0.0239 (2)	
C21	0.09231 (8)	0.93258 (16)	−0.16842 (5)	0.0259 (2)	
C22	−0.00938 (8)	0.94150 (16)	−0.18843 (5)	0.0297 (3)	
H22	−0.0506	0.9446	−0.1596	0.036*	
C23	−0.05075 (9)	0.94585 (17)	−0.25003 (6)	0.0327 (3)	
H23	−0.1200	0.9496	−0.2638	0.039*	
C24	0.01025 (10)	0.94466 (17)	−0.29052 (5)	0.0331 (3)	
C25	0.11065 (10)	0.93945 (18)	−0.27319 (6)	0.0353 (3)	
H25	0.1510	0.9410	−0.3025	0.042*	
C26	0.15114 (9)	0.93183 (17)	−0.21152 (5)	0.0311 (3)	
H26	0.2204	0.9260	−0.1984	0.037*	
C31	0.31015 (8)	0.57820 (16)	0.00173 (5)	0.0243 (2)	
C32	0.29576 (8)	0.39510 (16)	−0.02707 (5)	0.0279 (3)	
H32	0.2405	0.3764	−0.0593	0.033*	
C33	0.36021 (9)	0.24029 (17)	−0.00969 (5)	0.0308 (3)	
H33	0.3498	0.1162	−0.0294	0.037*	
C34	0.43955 (9)	0.27162 (17)	0.03683 (5)	0.0317 (3)	
C35	0.45767 (9)	0.44935 (18)	0.06604 (5)	0.0319 (3)	
H35	0.5135	0.4667	0.0980	0.038*	
C36	0.39282 (8)	0.60273 (17)	0.04799 (5)	0.0273 (3)	
H36	0.4049	0.7269	0.0675	0.033*	
C41	0.33623 (8)	1.17345 (18)	0.22707 (5)	0.0298 (3)	
C42	0.35335 (9)	1.1541 (2)	0.29036 (5)	0.0372 (3)	
H42	0.3345	1.0366	0.3078	0.045*	
C43	0.39754 (9)	1.3043 (2)	0.32801 (6)	0.0431 (3)	
H43	0.4086	1.2890	0.3709	0.052*	
C44	0.42550 (9)	1.4751 (2)	0.30357 (6)	0.0431 (3)	
H44	0.4572	1.5765	0.3295	0.052*	
C45	0.40733 (9)	1.4990 (2)	0.24114 (6)	0.0415 (3)	
H45	0.4256	1.6178	0.2241	0.050*	
C46	0.36256 (9)	1.35020 (19)	0.20337 (6)	0.0364 (3)	
H46	0.3495	1.3688	0.1605	0.044*	

### Atomic displacement parameters (Å^2^)

**Table:**

	*U*^11^	*U*^22^	*U*^33^	*U*^12^	*U*^13^	*U*^23^
F1	0.0717 (5)	0.0466 (5)	0.0227 (4)	0.0038 (4)	−0.0068 (4)	−0.0007 (3)
F2	0.0611 (5)	0.0429 (4)	0.0427 (5)	0.0271 (4)	0.0080 (4)	0.0095 (4)
O1	0.0557 (5)	0.0331 (5)	0.0312 (5)	−0.0031 (4)	0.0136 (4)	0.0058 (4)
O2	0.0426 (5)	0.0358 (5)	0.0272 (5)	0.0140 (4)	0.0067 (4)	−0.0025 (4)
C1	0.0259 (6)	0.0309 (6)	0.0255 (6)	0.0033 (5)	0.0079 (5)	0.0025 (5)
C2	0.0318 (6)	0.0336 (6)	0.0243 (6)	−0.0018 (5)	0.0047 (5)	0.0014 (5)
C3	0.0325 (6)	0.0347 (7)	0.0255 (6)	0.0022 (5)	0.0054 (5)	0.0037 (5)
C4	0.0400 (7)	0.0283 (6)	0.0429 (8)	0.0077 (5)	0.0100 (6)	−0.0037 (5)
C11	0.0248 (5)	0.0233 (5)	0.0225 (6)	−0.0028 (4)	0.0029 (4)	0.0001 (4)
C12	0.0271 (6)	0.0239 (6)	0.0260 (6)	−0.0015 (4)	0.0055 (5)	−0.0031 (5)
C13	0.0268 (6)	0.0236 (6)	0.0285 (6)	0.0016 (5)	0.0026 (5)	0.0016 (5)
C14	0.0259 (5)	0.0241 (6)	0.0244 (6)	−0.0033 (4)	0.0020 (5)	0.0005 (5)
C15	0.0271 (6)	0.0255 (6)	0.0248 (6)	−0.0006 (4)	0.0046 (5)	−0.0040 (5)
C16	0.0227 (5)	0.0217 (5)	0.0265 (6)	−0.0022 (4)	0.0032 (5)	−0.0002 (5)
C21	0.0310 (6)	0.0207 (5)	0.0242 (6)	−0.0009 (5)	0.0019 (5)	−0.0005 (4)
C22	0.0319 (6)	0.0273 (6)	0.0287 (6)	0.0017 (5)	0.0037 (5)	0.0008 (5)
C23	0.0333 (6)	0.0285 (6)	0.0318 (7)	0.0019 (5)	−0.0038 (5)	−0.0002 (5)
C24	0.0504 (8)	0.0235 (6)	0.0209 (6)	0.0016 (5)	−0.0034 (6)	0.0001 (5)
C25	0.0482 (8)	0.0315 (6)	0.0279 (7)	0.0009 (6)	0.0115 (6)	−0.0006 (5)
C26	0.0310 (6)	0.0312 (6)	0.0300 (7)	−0.0007 (5)	0.0040 (5)	−0.0018 (5)
C31	0.0250 (5)	0.0252 (6)	0.0236 (6)	0.0008 (4)	0.0073 (5)	0.0025 (4)
C32	0.0283 (6)	0.0276 (6)	0.0278 (6)	−0.0015 (5)	0.0060 (5)	−0.0003 (5)
C33	0.0390 (7)	0.0242 (6)	0.0321 (7)	0.0000 (5)	0.0139 (6)	−0.0002 (5)
C34	0.0371 (6)	0.0311 (6)	0.0296 (7)	0.0126 (5)	0.0129 (5)	0.0098 (5)
C35	0.0296 (6)	0.0396 (7)	0.0252 (6)	0.0042 (5)	0.0024 (5)	0.0031 (5)
C36	0.0296 (6)	0.0269 (6)	0.0252 (6)	0.0004 (5)	0.0051 (5)	−0.0009 (5)
C41	0.0271 (6)	0.0391 (7)	0.0222 (6)	0.0049 (5)	0.0028 (5)	−0.0009 (5)
C42	0.0399 (7)	0.0448 (8)	0.0257 (7)	0.0043 (6)	0.0041 (6)	0.0036 (6)
C43	0.0429 (7)	0.0596 (9)	0.0235 (7)	0.0066 (7)	−0.0005 (6)	−0.0054 (6)
C44	0.0379 (7)	0.0505 (9)	0.0377 (8)	−0.0004 (6)	0.0002 (6)	−0.0130 (6)
C45	0.0423 (7)	0.0415 (8)	0.0414 (8)	−0.0054 (6)	0.0098 (6)	−0.0032 (6)
C46	0.0415 (7)	0.0430 (7)	0.0243 (6)	−0.0015 (6)	0.0056 (5)	−0.0003 (5)

### Geometric parameters (Å, °)

**Table:**

F1—C24	1.3687 (13)	C23—C24	1.3658 (18)
F2—C34	1.3611 (13)	C23—H23	0.9500
O1—C1	1.2167 (14)	C24—C25	1.3722 (18)
O2—C12	1.3687 (13)	C25—C26	1.3843 (16)
O2—C4	1.4294 (14)	C25—H25	0.9500
C1—C2	1.4779 (16)	C26—H26	0.9500
C1—C11	1.5064 (15)	C31—C36	1.3926 (15)
C2—C3	1.3292 (16)	C31—C32	1.3980 (15)
C2—H2	0.9500	C32—C33	1.3860 (16)
C3—C41	1.4644 (17)	C32—H32	0.9500
C3—H3	0.9500	C33—C34	1.3719 (17)
C4—H4A	0.9800	C33—H33	0.9500
C4—H4B	0.9800	C34—C35	1.3730 (17)
C4—H4C	0.9800	C35—C36	1.3841 (16)
C11—C12	1.4012 (15)	C35—H35	0.9500
C11—C16	1.4065 (15)	C36—H36	0.9500
C12—C13	1.3849 (16)	C41—C46	1.3933 (17)
C13—C14	1.3915 (15)	C41—C42	1.3981 (16)
C13—H13	0.9500	C42—C43	1.3860 (18)
C14—C15	1.3928 (15)	C42—H42	0.9500
C14—C21	1.4893 (15)	C43—C44	1.375 (2)
C15—C16	1.3930 (15)	C43—H43	0.9500
C15—H15	0.9500	C44—C45	1.3819 (19)
C16—C31	1.4895 (15)	C44—H44	0.9500
C21—C26	1.3905 (16)	C45—C46	1.3832 (18)
C21—C22	1.3958 (15)	C45—H45	0.9500
C22—C23	1.3848 (16)	C46—H46	0.9500
C22—H22	0.9500		
			
C12—O2—C4	118.33 (9)	C23—C24—C25	123.23 (11)
O1—C1—C2	122.73 (11)	F1—C24—C25	118.43 (11)
O1—C1—C11	120.56 (10)	C24—C25—C26	117.78 (12)
C2—C1—C11	116.71 (10)	C24—C25—H25	121.1
C3—C2—C1	123.25 (11)	C26—C25—H25	121.1
C3—C2—H2	118.4	C25—C26—C21	121.26 (11)
C1—C2—H2	118.4	C25—C26—H26	119.4
C2—C3—C41	125.97 (11)	C21—C26—H26	119.4
C2—C3—H3	117.0	C36—C31—C32	118.00 (10)
C41—C3—H3	117.0	C36—C31—C16	122.32 (10)
O2—C4—H4A	109.5	C32—C31—C16	119.67 (10)
O2—C4—H4B	109.5	C33—C32—C31	121.53 (11)
H4A—C4—H4B	109.5	C33—C32—H32	119.2
O2—C4—H4C	109.5	C31—C32—H32	119.2
H4A—C4—H4C	109.5	C34—C33—C32	118.02 (11)
H4B—C4—H4C	109.5	C34—C33—H33	121.0
C12—C11—C16	119.11 (10)	C32—C33—H33	121.0
C12—C11—C1	117.76 (10)	F2—C34—C33	118.94 (10)
C16—C11—C1	123.03 (10)	F2—C34—C35	118.35 (11)
O2—C12—C13	123.90 (10)	C33—C34—C35	122.70 (10)
O2—C12—C11	114.63 (10)	C34—C35—C36	118.58 (11)
C13—C12—C11	121.37 (10)	C34—C35—H35	120.7
C12—C13—C14	119.77 (10)	C36—C35—H35	120.7
C12—C13—H13	120.1	C35—C36—C31	121.16 (11)
C14—C13—H13	120.1	C35—C36—H36	119.4
C13—C14—C15	119.05 (10)	C31—C36—H36	119.4
C13—C14—C21	120.42 (10)	C46—C41—C42	117.78 (11)
C15—C14—C21	120.46 (10)	C46—C41—C3	122.33 (11)
C14—C15—C16	121.98 (10)	C42—C41—C3	119.88 (11)
C14—C15—H15	119.0	C43—C42—C41	120.80 (12)
C16—C15—H15	119.0	C43—C42—H42	119.6
C15—C16—C11	118.62 (10)	C41—C42—H42	119.6
C15—C16—C31	119.24 (10)	C44—C43—C42	120.37 (12)
C11—C16—C31	122.14 (10)	C44—C43—H43	119.8
C26—C21—C22	118.68 (10)	C42—C43—H43	119.8
C26—C21—C14	121.47 (10)	C43—C44—C45	119.75 (12)
C22—C21—C14	119.81 (10)	C43—C44—H44	120.1
C23—C22—C21	120.55 (11)	C45—C44—H44	120.1
C23—C22—H22	119.7	C44—C45—C46	120.10 (13)
C21—C22—H22	119.7	C44—C45—H45	119.9
C24—C23—C22	118.47 (11)	C46—C45—H45	119.9
C24—C23—H23	120.8	C45—C46—C41	121.14 (12)
C22—C23—H23	120.8	C45—C46—H46	119.4
C23—C24—F1	118.33 (11)	C41—C46—H46	119.4
			
O1—C1—C2—C3	−9.33 (18)	C22—C23—C24—F1	179.77 (10)
C11—C1—C2—C3	171.15 (11)	C22—C23—C24—C25	−0.04 (18)
C1—C2—C3—C41	−179.84 (10)	C23—C24—C25—C26	1.09 (18)
O1—C1—C11—C12	122.58 (12)	F1—C24—C25—C26	−178.72 (10)
C2—C1—C11—C12	−57.89 (13)	C24—C25—C26—C21	−1.01 (18)
O1—C1—C11—C16	−53.63 (15)	C22—C21—C26—C25	−0.09 (17)
C2—C1—C11—C16	125.90 (11)	C14—C21—C26—C25	177.63 (11)
C4—O2—C12—C13	−16.63 (16)	C15—C16—C31—C36	136.15 (11)
C4—O2—C12—C11	166.88 (10)	C11—C16—C31—C36	−43.63 (15)
C16—C11—C12—O2	179.15 (9)	C15—C16—C31—C32	−42.56 (15)
C1—C11—C12—O2	2.78 (14)	C11—C16—C31—C32	137.65 (11)
C16—C11—C12—C13	2.56 (16)	C36—C31—C32—C33	1.30 (17)
C1—C11—C12—C13	−173.81 (10)	C16—C31—C32—C33	−179.93 (10)
O2—C12—C13—C14	−177.79 (10)	C31—C32—C33—C34	−0.23 (17)
C11—C12—C13—C14	−1.52 (16)	C32—C33—C34—F2	179.12 (10)
C12—C13—C14—C15	−1.23 (16)	C32—C33—C34—C35	−0.65 (17)
C12—C13—C14—C21	175.78 (10)	F2—C34—C35—C36	−179.38 (10)
C13—C14—C15—C16	2.99 (16)	C33—C34—C35—C36	0.40 (18)
C21—C14—C15—C16	−174.03 (10)	C34—C35—C36—C31	0.74 (17)
C14—C15—C16—C11	−1.93 (16)	C32—C31—C36—C35	−1.56 (16)
C14—C15—C16—C31	178.28 (10)	C16—C31—C36—C35	179.71 (11)
C12—C11—C16—C15	−0.84 (15)	C2—C3—C41—C46	2.43 (19)
C1—C11—C16—C15	175.33 (10)	C2—C3—C41—C42	−176.82 (12)
C12—C11—C16—C31	178.95 (10)	C46—C41—C42—C43	−1.74 (18)
C1—C11—C16—C31	−4.88 (16)	C3—C41—C42—C43	177.54 (11)
C13—C14—C21—C26	134.17 (12)	C41—C42—C43—C44	−0.12 (19)
C15—C14—C21—C26	−48.86 (16)	C42—C43—C44—C45	1.5 (2)
C13—C14—C21—C22	−48.15 (15)	C43—C44—C45—C46	−1.0 (2)
C15—C14—C21—C22	128.83 (12)	C44—C45—C46—C41	−0.9 (2)
C26—C21—C22—C23	1.17 (17)	C42—C41—C46—C45	2.25 (18)
C14—C21—C22—C23	−176.58 (11)	C3—C41—C46—C45	−177.01 (11)
C21—C22—C23—C24	−1.12 (17)		

### Hydrogen-bond geometry (Å, °)

**Table:**

*D*—H···*A*	*D*—H	H···*A*	*D*···*A*	*D*—H···*A*
C25—H25···O1^i^	0.95	2.41	3.3092 (15)	157
C44—H44···F2^ii^	0.95	2.55	3.2761 (15)	133

Symmetry codes: (i) *x*, −*y*+3/2, *z*−1/2; (ii) −*x*+1, *y*+3/2, −*z*+1/2.
